# The effects of ALDH2 Glu487Lys polymorphism on vasovagal syncope patients undergoing head-up tilt test supplemented with sublingual nitroglycerin

**DOI:** 10.1186/s12872-022-02901-5

**Published:** 2022-10-28

**Authors:** G Xia, JF Jin, Y Ye, XD Wang, B Hu, JL Pu

**Affiliations:** Department of Cardiology, Shanghai East Hospital, Tongji University School of Medicine, Shanghai, People’s Republic of China

**Keywords:** Vasovagal syncope, Nitroglycerin, Head-up tilt test, ALDH2 Glu487Lys polymorphism

## Abstract

**Background and objective:**

Head-up tilt test (HUTT) is clinically advantageous for diagnosing patients with vasovagal syncope (VVS). Nitroglycerin is mainly used as a stimulant during HUTT, and mitochondrial aldehyde dehydrogenase 2 (ALDH2) is involved in the metabolism of nitroglycerin (NTG). ALDH2 Glu487Lys polymorphism (ALDH2 rs671) is the most common variant in the East Asian population. This study aimed to assess the effects of ALDH2 rs671 on VVS patients undergoing HUTT supplemented with sublingual NTG (HUTT-NTG).

**Methods:**

Patients with recurrent VVS (at least 2 times) who were admitted to the syncope center of our hospital were enrolled. All VVS patients have undergone HUTT. The polymorphism of Glu487Lys gene of ALDH2 was measured by the DNA Microarray Chip Method. The results of HUTT-NTG of VVS patients with different ALDH2 genotypes were compared and their hemodynamic characteristics were assessed.

**Results:**

A total of 199 VVS patients were enrolled, including 101 patients in the ALDH2*1/*1 group and 98 patients in the ALDH2*2 group. Among patients undergoing HUTT-NTG, 70.3% of patients in the ALDH2*1/*1 group and 68.4% of patients in the ALDH2*2 group were positive, and the difference between the two groups was not statistically significant (*P* = 0.77). The proportions of VASIS I, VASIS II, and VASIS III were 40.6%, 8.9%, and 20.8% in the ALDH2*1/*1 group, respectively, and the corresponding proportions in the ALDH2*2 group were 36.7%, 11.2%, and 20.4%, respectively. There was no statistically significant difference between the two groups (*P* = 0.91). The hemodynamic characteristics of different genotypes in VVS patients undergoing HUTT-NTG were compared, and no statistically significant difference was found. The median time of syncopal episode occurred after NTG administration in the ALDH2*1/*1 group was 6 min (interquartile range [IQR]: 5.0–9.0), and it was 6.0 min in the ALDH2*2 group (IQR: 4.25–8.0, *P* = 0.64).

**Conclusion:**

ALDH2 Glu487Lys polymorphism did not affect the outcome of VVS patients undergoing HUTT-NTG, and no significant change in the hemodynamic characteristics of different genotypes was found.

## Introduction

Head-up tilt test (HUTT) plays an important role in the classification and formulation of treatment strategies for vasovagal syncope (VVS) [1]. The responses of positive tilt test patients were classified using the VAsovagal Syncope International Study (VASIS) criteria [2]. Treatment regimens vary among different subgroups [3]. HUTT is divided into two subtypes, including the baseline HUTT (B-HUTT) and drug-stimulated HUTT. Nitrates and isoproterenol are commonly used in the drug-stimulated HUTT [4]. At present, nitroglycerin (NTG) is the most frequently used stimulant drug for HUTT because of its rapidness, simplicity, and safety [5]. Aldehyde dehydrogenase 2 (ALDH2) is an important enzyme involved in the metabolism of NTG [6]. The enzymatic activity of ALDH2 significantly decreases after mutation. The encoding gene of ALDH2 is located on human chromosome 12 (12q24) and contains 13 exons [7]. Point mutation occurs at exon 12, resulting in the corresponding amino acid mutation from glutamate (Glu) to lysine (Lys), namely ALDH2 Glu504Lys (SNP rs671). ALDH2 rs671 polymorphism was commonly detected in the East Asian population (up to 54%) [8]. The enzymatic activity of heterozygous mutant ALDH2*1/*2 (Glu/Lys) decreased to 1/16, and homozygous mutant ALDH2*2/*2 (Lys/Lys) was almost lost [9].

However, the effects of ALDH2 polymorphism on patients receiving HUTT supplemented with sublingual NTG (HUTT-NTG) have still remained elusive. Hence, the present study aimed to investigate the influences of ALDH2 Glu487Lys polymorphism (ALDH2 rs671) on VVS patients undergoing HUTT-NTG.

## Methods

### Patient selection

This study complied with the principles of the Declaration of Helsinki and was approved by the Ethics Committee of Institutional Review Board of Shanghai East Hospital (2020–096).Patients with recurrent VVS who were admitted to Shanghai East Hospital (Shanghai, China) from January 1, 2020 to January 1, 2021 were enrolled. The exclusion criteria were as follows: 1) acute myocardial infarction; 2) severe valvular heart disease; 3) congenital heart disease; 4) myocarditis; 5) a history of coronary artery bypass grafting or aneurysm resection surgery; 6) II° or III° atrioventricular block (AVB); 7) sinoatrial block; 8) chronic cerebral arterial stenosis; 9) non-ischemic cardiomyopathy; 10) severe coronary stenosis (≥ 75%); 11) age ≥ 75 or < 18 years old; 12) the occurrence of VVS during B-HUTT.All participants showed their full intensions to our study.

### The diagnosis of vasovagal syncope

The diagnosis of vasovagal syncope complied with the 2018 ESC Guidelines for the Diagnosis and Management of Syncope [10]. The diagnosis of vasovagal syncope was based on the symptoms of the syncope episode. The following features suggested vasovagal syncope: 1) History of recurrent syncope episodes. 2). Triggers such as pain, fear, and unpleasant visual, auditory, taste, and/or olfactory stimuli. 3). Attacks in hot and/or crowded environments. 4). Prodromal symptoms such as pallor, sweating profusely, fatigue, nausea and vomiting. 5). Standing for a long time before the attack. The diagnosis of vasovagal syncope might be considered if the patient had the above characteristics,especially if cardiac syncope, orthostatic hypotension, carotid sinus syndrome and situational syncope were excluded.

#### HUTT process [11]

Patient preparation: coffee, tea, wine, and drugs affecting autonomic nervous function were stopped before the experiment. Patients had fasted for at least 4 h before the tests.

#### B-HUTT

The B-HUTT was performed in a quiet room under soft light at moderate temperature (20 ~ 25 ℃). Venous access was implemented before the B-HUTT. After 10 min of resting at supine position, patients were tilted to 70° in a head up, feet down position using an electronically operated tilt bed. During the experiment, blood pressure and heart rate were measured every 2 min, and electrocardiography (ECG) was performed continuously. In case of syncope occurrence, the trial was terminated immediately. Otherwise, the B-HUTT would last for 45 min.

#### HUTT supplemented with sublingual NTG (HUTT-NTG)

If positive result was not reached at the end of the B-HUTT, NTG (400 μg) was given sublingually in the tilt state, and the test was continued for further 20 min. During the test, blood pressure and heart rate were also measured every 2 min, and the test was terminated immediately when syncope occurred. The time of occurrence of symptoms was recorded and the test was considered positive. Syncope was classified as VASIS I, VASIS II, and VASIS III.

### Classification of syncope during HUTT [2]

Type 1 (VASIS I, mixed). Heart rate falls at the time of syncope, but the ventricular rate does not fall to less than 40 bpm, or falls to less than 40 bpm for less than 10 s with or without asystole of less than 3 s. Blood pressure falls before the heart rate falls.

Type 2 (VASIS II,cardioinhibition). Heart rate falls to a ventricular rate less than 40 bpm for more than 10 s.Dropping before or at the same time with the heart rate slows down.

Type 2A (VASIS IIA,cardioinhibition without asystole). Heart rate falls to a ventricular rate less than 40 bpm for more than 10 s, but asystole of more than 3 s does not occur.

Type 2B (VASIS IIB,cardioinhibition with asystole). Asystole occurs for more than 3 s.

Type 3( VASIS III, vasodepressor). SBP decreases to 60–80 mmHg or SBP or/and MAP decreases by 20–30 mmHg. Heart rate does not fall more than 10%, from its peak to the time of syncope.

#### DNA extraction and ALDH2 rs671 polymorphism analysis

Venous blood was extracted from patients with VVS. Genomic DNA was extracted from whole peripheral blood using the commercial whole blood genomic DNA extraction kit (Shanghai BaiO Technology Co., Ltd.,Shanghai,China). ALDH2 rs671 polymorphism was detected using ALDH2 Gene Detection Kit (Shanghai BaiO Technology Co., Ltd.,Shanghai,China.) which relies on DNA Microarray Chip Method. The operation process is carried out by professional inspectors according to the kit instructions.

#### Statistical analysis

In the present study, SPSS 22.0 software (IBM, Armonk, NY, USA) was used to perform the statistical analysis. The normal distribution of data was assessed using the Shapiro–Wilk test. Continuous variables with normal distribution were expressed as the mean ± standard deviation and compared using the Student’s t-test or one-way analysis of variance (ANOVA). Abnormally distributed data were presented as median and interquartile range (IQR) and compared using the non-parametric Mann–Whitney U test. Categorical variables were expressed as quantity and percentage and compared using the Chi-square test. *P* < 0.05 was considered statistically significant.

## Results

### Distribution of ALDH2 rs671 Glu487Lys polymorphism in VVS patients

Of the 241 patients diagnosed with VVS, 42 patients (17.4%) had syncope during the B-HUTT phase. A total of 199 VVS patients were enrolled. Genotype distributions did follow the Hardy–Weinberg equilibrium for ALDH2 gene in VVS patients [12]. Frequencies of ALDH2*1/*1, ALDH2*1/*2, and ALDH2*2/*2 genotypes were 50.8% (101/199), 43.2% (86/199), and 6.0% (12/199), respectively. As ALDH2 gene mutations mainly existed in the form of heterozygous and single mutation significantly affected the activity of ALDH2, heterozygous (ALDH2*1/*2) and homozygous (ALDH2*2/*2) mutations were grouped together (ALDH2*2) and compared with wild-type mutation (ALDH2*1/*1 group).

### Patients’ basic data

Of the 199 patients, 101 were in the ALDH2*1/*1 group and 98 were in the ALDH2*2 group. There was no significant difference between the two groups in age, sex ratio, hypertension, diabetes, and other chronic diseases. There were no significant differences in values of laboratory examination and left ventricular ejection fraction between the two groups (Table 1).

**Table 1:** Clinical data of VVS patients with different genotypes

Clinical data	ALDH2*1/*1 (*n* = 101)	ALDH2*2 (*n* = 98)	P
Male, n (%)	45 (44.6)	51 (52.0)	0.29
Age, years old	45.7 ± 12.6	48.3 ± 9.9	0.10
BMI, kg/m^2^	22.9 ± 1.41	23.2 ± 1.44	0.12
**Comorbidities**			
Hypertension, n (%)	11(10.9)	9(9.2)	0.69
Diabetes, n (%)	9(8.9)	10(10.2)	0.76
Drinking, n (%)	30(29.7)	31(31.6)	0.54
Current smoking, n (%)	34(33.7)	29(29.6)	0.77
**Laboratory data at discharge**			
WBC count, × 10^12^/L	6.5 ± 2.6	6.8 ± 2.4	0.47
Hb, g/dl	123.1 ± 11.7	126.4 ± 18.5	0.63
Platelet count, × 10^9^//L	205.5 ± 40.1	211.2 ± 47.5	0.52
eGFR, ml/min/1.73 m^2^	97.7 ± 6.6	96.5 ± 9.2	0.28
TG, mmol/L	1.74 ± 1.14	1.80 ± 1.19	0.73
TC, mmol/L	3.97 ± 0.88	3.98 ± 0.91	0.93
HDL-c, mmol/L	1.13 ± 0.27	1.12 ± 0.28	0.81
LDC-c, mmol/L	2.69 ± 0.87	2.66 ± 0.86	0.73
**LVEF, %**	57.8 ± 5.3	58.0 ± 4.0	0.75

*BMI* Body mass index, *WBC* White blood cell, *Hb* Hemoglobin, *eGFR* Estimated glomerular filtration rate (by modification of diet in renal disease (MDRD) equation), *TG* Total triglyceride, *TC* Total cholesterol, *HDL-C* High-density lipoprotein cholesterol, *LDL-C* Low-density lipoprotein cholesterol, *LVEF* left ventricular ejection fraction

### Comparison of HUTT results between the ALDH2*1/*1 and ALDH2*2 groups

The HUTT results were compared among different ALDH2 genotypes. In the ALDH2*1/*1 group, the proportion of HUTT-positive was 70.3%, while the proportion of HUTT-positive was 68.4% in the ALDH2*2 group, and the difference between the two groups was not statistically significant (Table 2, *P* = 0.77). In terms of subtype grouping of HUTT-positive patients, the proportions of VASIS I, VASIS II, and VASIS III between the two groups were not statistically significant difference (Table 3, *P* = 0.91).

**Table 2:** Comparison of the results of HUTT stimulated by NTG among different genotypes

HUTT results	ALDH2*1/*1 (*n* = 101)	ALDH2*2 (*n* = 98)	P
Negative, n (%)	30 (29.7)	31 (31.6)	0.77
Positive, n (%)	71 (70.3)	67 (68.4)	

*HUTT* Head-up tilt test

**Table 3:** Comparison of VVS subtypes among different genotypes

Type of VVS	ALDH2*1/*1 (*n* = 101)	ALDH2*2 (*n* = 98)	P
Negative, n (%)	30 (29.7)	31 (31.6)	0.91
VASIS I (mixed type), n (%)	41 (40.6)	36 (36.7)	
VASIS II (cardiac inhibitory type), n (%)	9 (8.9)	11 (11.2)	
VASIS III (vascular inhibitory type), n (%)	21 (20.8)	20 (20.4)	

*VASIS* VAsovagal Syncope International Study

### Comparison of hemodynamics and syncope time during HUTT-NTG between the ALDH2*1/*1 and ALDH2*2 groups

**Fig. 1:**
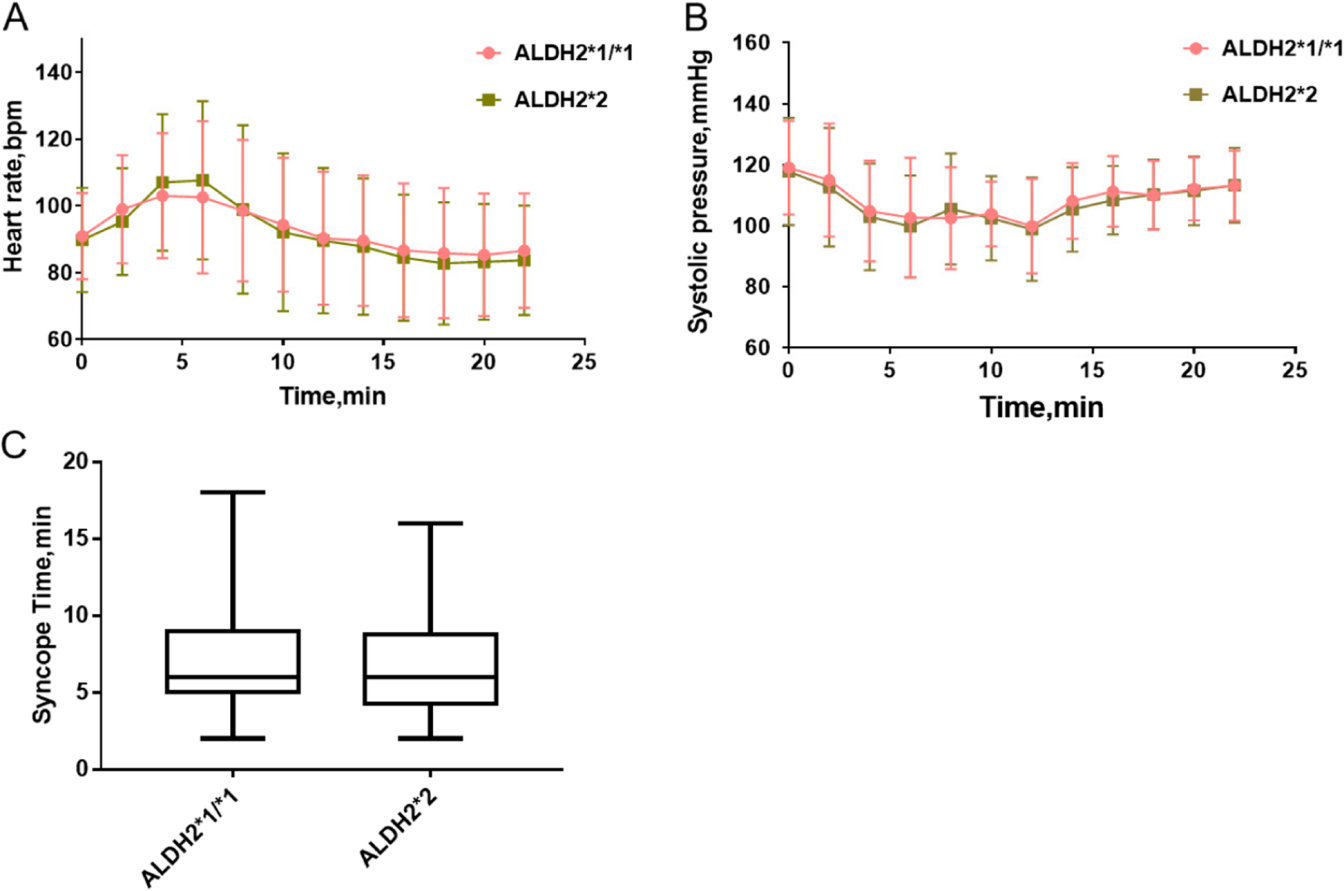
Hemodynamics and syncope time during HUTT-NTG in the ALDH2*1/*1 and ALDH2*2 groups. **A** HR changed during HUTT-NTG. **B** Changes in systolic blood pressure during HUTT-NTG. **C** Time box diagram of syncopal episodes during HUTT-NTG

Changes in heart rate (HR) and systolic blood pressure (SBP) during HUTT-NTG were compared between the two groups. Shapiro–Wilk test showed that HR and SBP data were abnormal distribution data (*P* < 0.01). But the histograms of the data distribution revealed that the data was approximately normally distributed. In the process of data analysis, non-parametric test and t-test were used respectively, and no significant statistical difference was found. Therefore, in order to include more experimental data, mean ± standard deviation was selected to display these data.There was a significant increase in heart rate in the early-stage of HUTT-NTG and a decrease due to syncope, while there was no statistically significant difference between the two groups (Fig. 1A). There was a slight decrease in SBP after NTG administration, and a further significant decrease due to syncope, whereas the difference between the two groups was not statistically significant (Fig. 1B). The time of syncopal episode in HUTT-positive patients was compared between the two group. The time of taking NTG under the tongue as the starting time was defined as 0 min. The normality of the syncopal episode time was assessed by the Shapiro–Wilk test.The significance of Shapiro–Wilk test was *P* < 0.01, indicating that the distribution of syncope did not conform to the normal distribution. The median time of syncope occurred after NTG administration in the ALDH2*1/*1 group was 6 min (IQR: 5.0–9.0), and it was 6.0 min in the ALDH2*2 group (IQR: 4.25–8.0, *P* = 0.64, Fig. 1C).

## Discussion

Although ALDH2 is typically involved in the metabolism of NTG, ALDH2 mutations did not affect the outcome of patients with VVS undergoing HUTT-NTG in the present study.

The HUTT was originally proposed to study the physiological compensatory response to orthostatic stress [13]. Kenny et al. first applied HUTT in clinical trials in 1986, and found that unexplained syncope could be induced [14]. Thereafter, a large number of studies have confirmed that HUTT is a safe and effective diagnostic method.

HUTT consists of B-HUTT and drug-stimulated HUTT. Our study found that the proportion of syncope occurring in B-HUTT was 17.4%, while the proportion in drug-induced HUTT was 57.3%, which was similar to the results of previous studies [15]. Syncope often occurs in the drug-induced phase during HUTT examination.

**Fig. 2:**
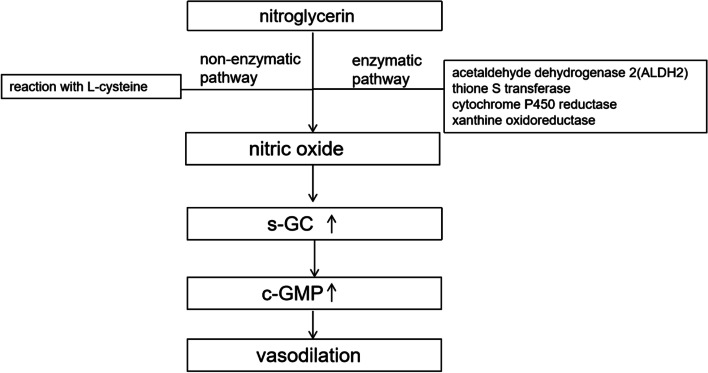
A simple diagram of nitroglycerin metabolism

NTG and isoproterenol are commonly used as stimulants during drug-induced HUTT. The modern view is that isoproterenol directly excites the sympathetic nerve during HUTT, while nitroglycerin indirectly excites the sympathetic nerve through its hypotensive effect [16]. A meta-analysis showed that nitroglycerin was more sensitive than isoproterenol in inducing HUTT [17]. Nitroglycerin is more commonly used due to its convenience and safety. The mechanism of nitroglycerin in lowering blood pressure during HUTT is still controversial. Small dose of nitroglycerin is traditionally considered to be effective venodilators, leading to a decrease in ventricular preload and cardiac output (CO), with little effect on arterial resistance [18]. This effect of nitroglycerin is generally considered to be the main mechanism of nitroglycerin-induced vasovagal syncope. However, other studies have found that nitroglycerin-induced systemic hypotension in vasovagal response is a reduction in systemic vascular resistance (SVR) [19]. Whether reducing preload or systemic arterial pressure, the main effect of NTG is achieved through its metabolite-NO. NO activates guanylate cyclase (sGC), which increases tissue production of cGMP. cGMP then activates cGMP-dependent protein kinase (cGK) that mediates vasodilation by phosphorylating proteins that regulate intracellular calcium concentration and cytoskeleton (Fig. 2). Moreover, some studies have found that the level of NO changes in VVS patients during HUTT, suggesting that NO itself may be involved in the process of HUTT [20, 21].

Several enzymes have been found to be involved in the metabolism of nitroglycerin to NO, including acetaldehyde dehydrogenase 2, thione S transferase, cytochrome P450 reductase, old flavase, and xanthine oxidoreductase.As Chen et al. [22] reported that ALDH2 plays an important role in the bio-activation of NTG, more and more studies have shown that ALDH2 is one of the most important enzymes in nitroglycerin metabolism to NO. Studies of the effects of ALDH2 on nitroglycerin mainly focused on coronary artery disease, such as myocardial infarction, coronary heart disease, and coronary artery spasm. Hirofumi Yasue et al. found ALDH2*2 attenuated GTN response and exacerbated GTN tolerance in coronary spasm patients [23]. Li Jin et al. found that the presence of the Lys504 allele of ALDH2 contributes in part to the failure of the efficacious response to nitroglycerin in coronary heart disease [24].

However, the effect of ALDH2 gene polymorphism on the outcome of HUTT-NTG in VVS patients remains unclear. We tried to explore this question. Frequencies of ALDH2*1/*1, ALDH2*1/*2, and ALDH2*2/*2 genotypes were 50.8% (101/199), 43.2% (86/199), and 6.0% (12/199) in VVS patients in the present study, which are similar to the rates previously reported in the general Chinese population [12]. The present study found no significant differences in the incidence of syncope and subtypes among ALDH2 genotypes during the HUTT-NTG, suggesting that ALDH2 genotypes did not influence the outcome of VVS patients undergoing HUTT-NTG. There was no significant difference in the median time of syncope after NTG administration between the two groups. This phenomenon may be explained by the involvement of other enzymes and other bypass pathways in the bioconversion process of NTG, and the activation or upregulation of other metabolic pathways of NTG may occur in the presence of low or complete loss of ALDH2 activity.. In addition, in vitro and in vivo studies also suggested that NTG may not be completely dependent on enzymatic pathway, while it may act through the non-guanylate cyclase pathway [25].

Our results suggest that ALDH2 gene polymorphism does not affect the outcome of HUTT-NTG in VVS patients. Whether nitroglycerin can be used to induce HUTT in ALDH2 mutant population may be an easily overlooked question.But it's also a question that should be considered since there is such a high proportion of heterozygous and homozygous mutations in the population. Our study provides an answer to that question.

The study had its limiations. First,this was a single-center, small sample size study. Further multicenter, large-scale study should be conducted to verify the results.Second, the results of HUTTs performed on the same VVS patient for several times might not be completely consistent, which might have a certain influence on the results.

## Conclusion

To sum up, the present study did not find differences in HUTT-NTG results and types of VVS among different ALDH2 genotypes. There was no significant difference in hemodynamic characteristics between the two ALDH2 genotypes in VVS patients undergoing HUTT-NTG.

## Data Availability

All data generated or analysed during this study are included in this published article. The original data can be obtained by contacting the corresponding author.

## Abbreviations

HUTT: Head-up tilt test
VVS: Vasovagal syncope
ALDH2: Aldehyde dehydrogenase 2
NTG: Nitroglycerin
HUTT-NTG: HUTT supplemented with sublingual NTG
IQR: Interquartile range
B-HUTT: Baseline HUTT
VASIS: VAsovagal Syncope International Study
HR: Heart rate
SBP: Systolic blood pressure
CO: Cardiac output
SVR: Systemic vascular resistance
